# Association of Continuous Assessment of Step Count by Remote Monitoring With Disability Progression Among Adults With Multiple Sclerosis

**DOI:** 10.1001/jamanetworkopen.2019.0570

**Published:** 2019-03-15

**Authors:** Valerie J. Block, Riley Bove, Chao Zhao, Priya Garcha, Jennifer Graves, Andrew R. Romeo, Ari J. Green, Diane D. Allen, Jill A. Hollenbach, Jeffrey E. Olgin, Gregory M. Marcus, Mark J. Pletcher, Bruce A. C. Cree, Jeffrey M. Gelfand

**Affiliations:** 1 Weill Institute for Neurosciences, Department of Neurology, University of California, San Francisco; 2 Department of Ophthalmology, University of California, San Francisco; 3 Associate Editor, *JAMA Neurology*; 4 Department of Physical Therapy and Rehabilitation, University of California, San Francisco; 5 Department of Physical Therapy and Rehabilitation, San Francisco State University, San Francisco, California; 6 Department of Epidemiology and Biostatistics, University of California, San Francisco

## Abstract

**Importance:**

Disability measures in multiple sclerosis (MS) fail to capture potentially important variability in walking behavior. More sensitive and ecologically valid outcome measures are needed to advance MS research.

**Objectives:**

To assess continuous step count activity remotely among individuals with MS for 1 year and determine how average daily step count is associated with other measures of MS disability.

**Design, Setting, and Participants:**

In a prospective longitudinal observational cohort study, 95 adults with relapsing or progressive MS who were able to walk more than 2 minutes with or without an assistive device were recruited between June 15, 2015, and August 8, 2016, and remotely monitored in their natural environment for 1 year. Patients were excluded if they had a clinical relapse within 30 days or comorbidity contributing to ambulatory impairment. Longitudinal analysis was performed from October 2017 to March 2018. Revised analysis was performed in December 2018.

**Intervention:**

Activity monitoring of step count using a wrist-worn accelerometer.

**Main Outcomes and Measures:**

Average daily step count compared with in-clinic assessments and patient-reported outcomes.

**Results:**

Of the 95 participants recruited (59 women and 36 men; mean [SD] age, 49.6 [13.6] years [range, 22.0-74.0 years]), 35 (37%) had progressive MS, and the median baseline Expanded Disability Status Scale score was 4.0 (range, 0-6.5). At 1 year, 79 participants completed follow-up (83% retention). There was a modest reduction in accelerometer use during the 1 year of the study. A decreasing average daily step count during the study was associated with worsening of clinic-based outcomes (Timed 25-Foot Walk, β = −13.09; *P* < .001; Timed-Up-and-Go, β = −9.25; *P* < .001) and patient-reported outcomes (12-item Multiple Sclerosis Walking Scale, β = −17.96; *P* < .001). A decreasing average daily step count occurred even when the Expanded Disability Status Scale score remained stable, and 12 of 25 participants (48%) with a significant decrease in average daily step count during the study did not have a reduction on other standard clinic-based metrics. Participants with a baseline average daily step count below 4766 (cohort median) had higher odds of clinically meaningful disability (Expanded Disability Status Scale score) worsening at 1 year, adjusting for age, sex, and disease duration (odds ratio, 4.01; 95% CI, 1.17-13.78; *P* = .03).

**Conclusions and Relevance:**

Continuous remote activity monitoring of individuals with MS for 1 year appears to be feasible. In this study, a decreasing average daily step count during a 1-year period was associated with worsening of standard ambulatory measures but could also occur even when traditional disability measures remained stable. These results appear to support the prospect of using the average daily step count as a sensitive longitudinal outcome measure in MS and as a clinically relevant metric for targeted intervention.

## Key Points

**Question:**

Is remote monitoring of continuous activity for 1 year among individuals with multiple sclerosis associated with other measures of ambulatory disability?

**Findings:**

In this longitudinal cohort study, 95 individuals with multiple sclerosis were asked to wear accelerometers for 1 year; at baseline, a low step count was associated with increased risk of clinical worsening at 1 year. Step count reduction during the study was associated with worsening of clinic-based and patient-reported outcomes and occurred even when conventional disability measures remained stable.

**Meaning:**

Remote step count monitoring might represent a sensitive longitudinal outcome measure with direct implications for multiple sclerosis clinical trials and care.

## Teaser

This longitudinal cohort study assesses how remote monitoring of continuous step count activity for 1 year among individuals with multiple sclerosis is associated with other measures of multiple sclerosis disability.

## Introduction

Multiple sclerosis (MS) is a leading cause of nontraumatic disability in the developed world, with walking impairment a major contributing factor.^1,2,3^ Despite its widespread use in clinical trials, the Expanded Disability Status Scale (EDSS), a rater-based categorical measure of disability in patients with MS, has limitations with reliability and insufficient sensitivity as an outcome measure for small but still clinically meaningful changes in ambulatory function.^4,5^ For example, a person who walks 1.6 km (1 mile) a day with a cane and a person who can barely walk around his or her home using a cane (but past a minimal distance threshold of 120 m) are both assigned the same EDSS score (6.0; based on need for unilateral assistance) despite a clear disparity in ambulatory disability functioning. Clinic-based performance measures, such as timed walks, provide informative snapshots about ambulatory function but do not capture performance fluctuations in the natural environment.^6^ Patient-reported outcomes provide valuable insights but are subject to recall bias and may be associated with variable perception of similar deficits. Therefore, more objective and ecologically valid outcome measures of walking are needed to advance MS research and help make clinical trials more efficient by having more sensitive outcome measures to improve power. Other neurologic disorders that affect walking pose similar methodological challenges to MS research and could also benefit from better ambulatory outcome metrics.

Remote activity monitoring using wearable accelerometers records how much a person actually moves in his or her daily life. By providing a clinically meaningful metric of physical activity in the natural environment,^2^ remote accelerometry may be advantageous as an outcome measure and has the potential to inform clinical care and neurologic rehabilitation.

A previous study demonstrated the validity of 4 weeks of continuous remote activity monitoring of individuals with MS.^7^ In this study, we aim to show that continuous year-long remote activity monitoring using an accelerometer in a prospective cohort of individuals with MS is feasible, that change in the average daily step count is associated with validated disability outcomes, and that the average daily step count is a valid and useful outcome measurement tool.

## Methods

### Participants

Participants with relapsing or progressive MS as defined by 2010 International Panel criteria^8^ were recruited from the University of California, San Francisco Multiple Sclerosis Center between June 15, 2015, and August 8, 2016, into a prospective cohort study: FITRiMS (Fitbit Remote monitoring in MS). We included participants who were older than 18 years, were able to walk for at least 2 minutes (with or without an assistive device; limiting the cohort to those with an EDSS score of <7.0) at study entry, had access to WiFi internet, and had not experienced a relapse during the 30 days prior to enrollment. Exclusion criteria included any musculoskeletal or cardiovascular comorbidities that could affect ambulatory function or the participant’s ability to consent or follow study instructions. To ensure that study participants were representative of the full range of disability in ambulating patients, we block recruited based on EDSS score (0-1.5 indicating minimal disability; 2-3.5, mild disability; 4, mild ambulatory disability; 4.5-5.5, moderate ambulatory disability; 6, need for unilateral support; and 6.5, bilateral support needed to walk). The University of California, San Francisco Institutional Review Board approved the study protocol, and all participants provided written informed consent. Baseline cross-sectional findings at 4 weeks were previously reported.^7^ This study followed the Strengthening the Reporting of Observational Studies in Epidemiology (STROBE) reporting guidelines. Longitudinal analysis was performend from October 2017 to March 2018. Revised analysis was performed in December 2018.

### Study Procedures

At the baseline visit, participants were asked to wear a commercially available activity monitor (Fitbit Flex; Fitbit Inc)^7^ on their nondominant wrist for 1 year. Participants were instructed to continue their normal daily lives, and step count was continuously monitored. Lost or damaged devices were replaced. This study leveraged a platform developed by the University of California, San Francisco Health eHeart study^9^ for electronic consent as well as collection and storage of the activity monitoring data that uses an application programming interface that pulls data from Fitbit.com.

At baseline and 1-year follow-up, investigators evaluated the following clinic-based measures: EDSS, Timed 25-Foot Walk (T25FW),^2^ Timed-Up-and-Go (TUG),^10^ and the 2-minute walk test.^11^ The EDSS was performed by a trained MS neurologist (R.B., J.G., A.R.R., A.J.G., B.A.C.C., and J.M.G.). Performance-based outcomes were evaluated by a physical therapist (V.J.B.). For 5 participants unable to return to clinic for the 1-year follow-up (owing to geographic factors and personal issues), a tele-EDSS assessment^12^ using a previously validated instrument was conducted by a study MS neurologist (R.B. and J.M.G.) via secure video conferencing (Zoom platform; Zoom Video Communications). Participants completed self-reported questionnaires (12-item Multiple Sclerosis Walking Scale [MSWS-12],^13^ Modified Fatigue Index,^14^ Mental Health Inventory,^15^ Bladder and Bowel Control Scales,^14^ MOS [Medical Outcomes Study] Pain Effects Scale,^16^ and the World Health Organization Disability Assessment Schedule 2.0^17^) addressing MS-related disability and symptoms via a secure website (REDCap) at study entry (baseline), 1.5, 3, 6, 9, and 12 months. A link to the survey was sent at the follow-up times, and automatic reminders were sent every 3 days thereafter if the questionnaires were not completed. As a proactive study management strategy, step count activity on the accelerometer was monitored on a weekly basis, and email reminders to wear or synchronize the device were sent to participants who recorded no steps per day for more than 5 days at a time.

### Statistical Analysis

Guided by previous research,^7,18,19^ we defined a valid day as one in which participants took 128 steps or more, and a valid week as one that had 3 or more valid days. Because the average daily step count was the focus of measurement for this study, and our primary interest was to determine how established metrics are associated with the average daily step count and the change in the average daily step count over time, we used the average daily step count as our primary outcome measure. To account for some missing data and to be inclusive of all participants’ average daily step count, we used weighted mean values (each individual’s average daily step count was multiplied by the portion of the number of valid days he or she provided for the analysis). The baseline average daily step count was defined as the average daily step count averaged for the first 4 consecutive valid weeks. The 1-year average daily step count was the mean value for month 12 (52nd week, ±1 month).

A clinically meaningful change in disability (EDSS score) was defined as a 1.5-point change when the baseline EDSS score was from 0.0 to 1.0, was defined as a 1.0-point change when the baseline EDSS score was from 1.5 to 5.0, and was defined as a 0.5-point change when the baseline EDSS score was from 5.5 to 6.5.^20^ A 20% change in the T25FW was considered clinically meaningful.^21^ Scores of 11.5 seconds or more on the TUG were used as a cutoff for change in mobility and balance, dichotomizing the cohort into participants’ balance as impaired (≥11.5 seconds) or stable or unimpaired (<11.5 seconds).^22^ For the MSWS-12, an 8-point decrease in total score was considered a clinically meaningful improvement.^23^

To determine any significant differences in baseline data comparing those who were lost to follow-up or withdrew with those who completed the study, we performed a *t* test or χ^2^ analysis. All *P* values were from 2-sided tests and results were deemed statistically significant at *P* < .05. The Pearson χ^2^ test with the Yates continuity correction was used to determine the proportion of device use between participants with progressive MS and participants with relapsing MS. The mean number of consecutive days of device use between MS subtypes was evaluated using the *t* test.

We defined worsening of the average daily step count during the 1-year study as a significantly negative slope (*P* < .05). Preliminary data from a prior cross-sectional study of patients with relapsing-remitting MS postulated that a clinically meaningful change in average daily step count could be defined as 800 steps or more,^24^ but because our study is longitudinal and also includes patients with progressive MS, we elected to use this more inclusive definition of a negative slope for our analysis.

To determine which clinic-based outcome variables (grouped by EDSS, T25FW, and TUG scores) were significantly associated with the rate of change in average daily step count during the year, we used the Wald test. This test can be used for a multitude of different models, including those with binary variables or continuous variables.

Linear regression models were used to evaluate associations between changes in clinic-based measures (EDSS group, T25FW, and TUG) and changes in the average daily step count over time. Bonferroni corrections were conducted for multiple comparisons analysis. Linear regression models were used to compare baseline disability (EDSS group at study entry, as well as Functional System Scores) with the rate of change (slope) in average daily step count taken during the year. *P* values are based on SEs.

Multivariable linear regression models with random effects including time as an interaction term were used to analyze continuous, repeated-measures data (change in average daily step count over time) between sex and MS type. We adjusted for sex, age, and disease duration when applicable using R, version 3.5.0, package lme4 (R Foundation for Statistical Computing). Logistic regression was used to determine whether a participant’s baseline average daily step count was associated with a change in EDSS score (or Functional System Score) during the year.

To evaluate any difference in average daily step count (weighted and unweighted mean values) taken during the year by participants with progressive or relapsing MS, we performed linear mixed modeling. To determine the association of the change (decrease) in average daily step count during the year with either sex or mental health, and within individual disease categories, we performed linear mixed modeling. To determine how sampling with shorter measurement periods of the average daily step count compared with continuous monitoring during the year, the difference in slopes (change in average daily step count) for shorter durations was analyzed systematically, eliminating 1 month at a time and compared with 1-year data.

Odds ratios (ORs) were calculated to determine the association between changes in patient-reported outcomes (Modified Fatigue index, MSWS-12, Mental Health Inventory, MOS Pain Effects Scale, World Health Organization Disability Assessment Schedule 2.0, and Bladder and Bowel Control Scales) and change in average daily step count during the year, dichotomizing the average daily step count to worsening (β < 0) and improvement (β > 0) as the outcome, with *P* < .05 considered statistically significant. For all regression lines, 95% CIs were calculated from the regression model. The open-source programming language R (R Foundation for Statistical Computing) was used for all analyses.

## Results

### Feasibility of Continuous Remote Activity Monitoring for 1 Year of Individuals With MS

Ninety-five individuals with MS were prospectively recruited. At 1 year, 79 individuals completed the study (83% retention). Nine participants withdrew from the study (personal reasons [n = 5], unable to sync device with personal electronics [n = 1], relocation [n = 1], and cutaneous sensitivity [n = 2]). Seven participants were lost to follow-up. Of the 95 individuals in the retained cohort, 60 (63%) had relapsing MS, and 35 (37%) had progressive MS; 59 participants were women, and 36 participants were men, the mean (SD) age was 49.6 (13.6) years (range, 22.0-74.0 years), the median disease duration was 13.0 years (interquartile range, 5.0-20.5 years), and the median baseline EDSS score was 4.0 (range, 0-6.5) (eTable 1 in the Supplement). No significant differences in baseline data were observed between those who were lost to follow-up or withdrew from the study and those who completed the study (eTable 2 in the Supplement). Implementing full valid average daily step count data for the year resulted in sample size reductions for analysis of the T25FW (n = 65) and TUG (n = 61). Data from the TUG, T25FW and 2-minute walk test results were not available for the 5 participants whose 1-year follow-up visit was conducted via video-conferencing (Table).^24^ We retrieved 9 T25FW times measured in the clinic via the electronic medical records.

**Table.** Summary of Clinic-Based and Average Daily Step Count Outcomes

Outcome	Baseline	At 1 y	Absolute Change, No./Total No. (%)			Clinically Meaningful Change, No./Total No. (%)^a^		
			Worsened	Improved	No Change	Worsened	Improved	No Change
**EDSS**								
All patients, No.	95	79	NA	NA	NA	NA	NA	NA
Median score (IQR)	4.0 (2.5-6.0)	4.5 (2.5-6.0)	27/79 (34)	23/79 (29)	29/79 (37)	21/79 (27)	15/79 (19)	43/79 (54)
Patients with relapsing MS, No.	60	49	NA	NA	NA	NA	NA	NA
Median score (IQR)	3.0 (2.0-5.0)	3.0 (2.0-5.0)	14/49 (29)	1849 (37)	17/49 (35)	11/49 (22)	13/49 (27)	25/49 (51)
Patients with progressive MS, No.	35	30	NA	NA	NA	NA	NA	NA
Median score (IQR)	6.0 (4.0-6.5)	6.0 (4.5-6.5)	13/30 (43)	5/30 (17)	12/30 (40)	10/30 (33)	2/30 (6.7)	18/30 (60)
**T25FW, s**								
All patients, No.	95	70	NA	NA	NA	NA	NA	NA
Mean (SD)	7.31 (5.61)	7.51 (4.63)	34/70 (49)	12/70 (17)	24/70 (34)	23/70 (33)	3/70 (4)	44/70 (63)
All patients, No.^b^	95	65	NA	NA	NA	NA	NA	NA
Mean (SD)^b^	NA	NA	30/65 (46)	12/65 (18)	23/65 (35)	21/65 (32)	3/65 (5)	41/65 (63)
**TUG, s**								
All patients, No.	95	65	NA	NA	NA	NA	NA	NA
Mean (SD)	11.94 (11.22)	10.91 (7.39)	20/65 (31)	27/65 (42)	18/65 (28)	20/65 (31)	45/65 (69)^c^	NA
All patients, No.^b^	95	61	NA	NA	NA	NA	NA	NA
Mean (SD)^b^	NA	NA	19/61 (31)	25/61 (41)	17/61 (28)	19/61 (31)	41/61 (70)	NA
**Average Daily Step Count**								
All patients, No.	95	55	NA	NA	NA	NA	NA	NA
Median (IQR)	4766.2 (2986.7-7448.0)	4989.6 (2802.3-7409.1)	30/91 (33)^d^	8/91 (9)^d^	53/91 (58)^d^	20/55 (36)^b^	14/55 (26)^b^	21/55 (38)^b^
Patients with relapsing MS, No.	60	36	NA	NA	NA	NA	NA	NA
Median (IQR)	5540.1 (3862.1-8627.5)	6082.3 (4159.6-8205.4)	16/60 (27)	7/60 (12)	36/60 (60)	13/36 (36)^b^	9/36 (25)^b^	14/36 (39)^b^
Patients with progressive MS, No.	35	19	NA	NA	NA	NA	NA	NA
Median (IQR)	3409.5 (2199.0-5461.6)	2806.4 (2028.6-6300.4)	14/35 (40)	1/35 (3)	17 (49)	7/19 (37)^b^	5/19 (26)^b^	7/19 (37)^b^

Abbreviations: EDSS, Expanded Disability Status Scale; IQR, interquartile range; MS, multiple sclerosis; NA, not applicable; T25FW, Timed 25-Foot Walk; TUG, Timed-Up-and-Go.

^a^ Clinically meaningful disability change (as measured by the EDSS) was defined as a 1.5-point change when baseline EDSS score was from 0.0 to 1.0, a 1.0-point change when baseline EDSS score was from 1.5 to 5.0, and a 0.5-point change when baseline EDSS score was from 5.5 to 6.5. A 20% change was considered clinically meaningful for T25FW. Changes in TUG scores of 11.5 seconds or higher were used as the cutoff for change in mobility and balance, dichotomizing the cohort into participants with worsened (≥11.5 seconds) or improved or no change (<11.5 seconds). For the TUG, improved and no change were combined to allow for adequate sample size. Clinically meaningful change in average daily step count was defined as 800 or more steps as previously suggested in a cross-sectional cohort in patients with relapsing-remitting MS.^24^

^b^ After quality control for 1 full valid year of average daily step count measured by the accelerometer.

^c^ Improved or no change.

^d^ Based on quality control over time.

**Figure 1.**
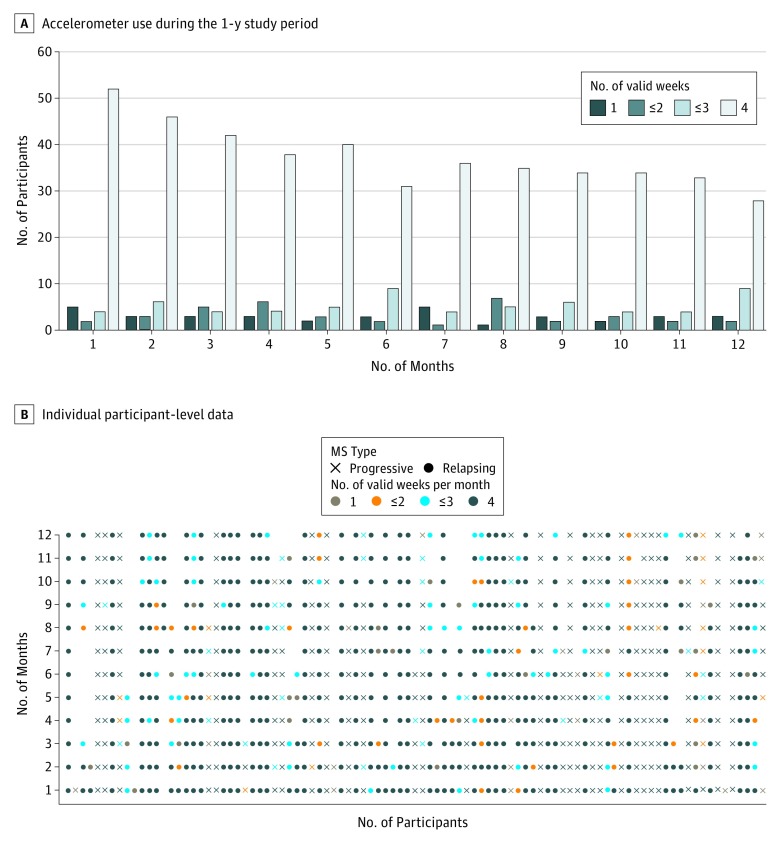
Accelerometer Use in a Prospective Research Cohort of Individuals With Multiple Sclerosis (MS) During a 1-Year Period A, Accelerometer use during the 1-year study period. Each bar represents the number of participants with valid Fitbit data (≥128 average daily steps per day, ≥3 days per week) during the 1-year study. B, Individual participant-level data by month, depicting periods of use and disuse of the wrist-worn accelerometer for 12 months. Each dot represents valid accelerometer data collected for 1 individual participant for that month.

The cohort as a whole provided a mean of 3 valid weeks of average daily step counts per month, and device use gradually decreased during the year (Figure 1A). The longest consecutive time the device was worn was not significantly different between participants with progressive MS (median, 77.5 days; mean, 104.87 days) and relapsing MS (median, 93 days; mean, 129.9 days; *P* = .27). There were no differences in baseline characteristics from month 1 to month 11 when comparing participants who had more than 1 week per month of usable data vs those who had only 1 week per month of usable data. However, during month 12, participants with only 1 week per month of valid data had poorer clinic-based and patient-reported measures at baseline compared with those who had more than 1 week per month of valid data (longer mean TUG times [26.6 vs 11.6 minutes; *P* = .02], poorer bladder function [mean Bladder Control Scale score: 11.5 vs 5.2; *P* = .02], more pain [mean MOS Pain Effects Scale score: 18.5 vs 12.4; *P* = .03], and greater disability [mean World Health Organization Disability Assessment Schedule 2.0 score: 19.5 vs 9.7; *P* = .01]). Figure 1B shows individual participant-level data for each month. There was no significant difference in the proportion of device use and disuse between participants with progressive MS and participants with relapsing MS (progressive MS, 26 of 30 [33% of all 79 patients]; and relapsing MS, 38 of 49 [48% of all 79 patients]; *P* = .48) nor between the mean consecutive days of device use (progressive MS, 105 days vs relapsing MS, 130 days; *P* = .26). Twenty-two devices were replaced (17 because of loss and 5 because of malfunction).

### Change in Average Daily Step Count and EDSS Score

**Figure 2.**
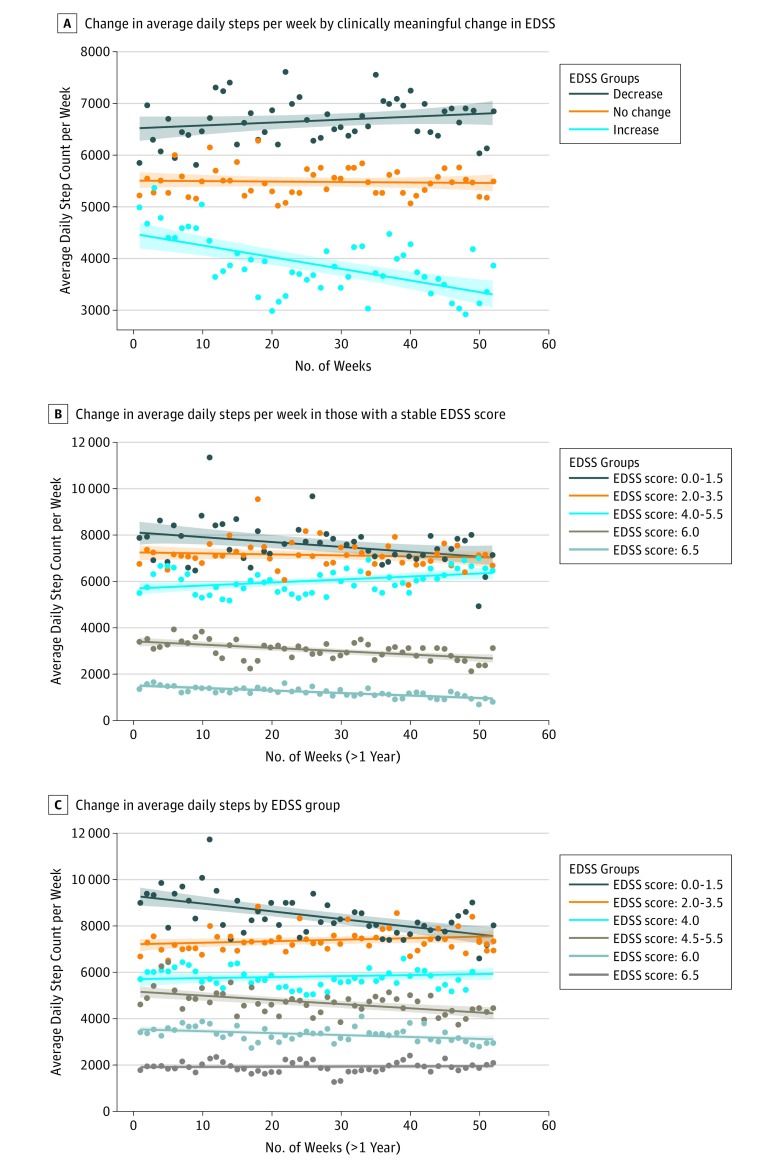
Change in Average Daily Step Count Stratified by Expanded Disability Status Scale (EDSS) Score A, Change in continuous average daily step count per week during a 1-year period, stratified by clinically meaningful change in EDSS score. B. Change in average daily step count per week during a 1-year period among individuals with a stable EDSS score (groups with EDSS scores of 4.0 and 4.5-5.5 were combined to achieve an adequate sample size). C, Change in average daily step count per week during the 1-year study by EDSS group. In each panel, the shaded area represents the 95% CIs for the regression line, and each point reflects the daily step count averaged per week (using weighted means) for individuals who had clinically meaningful worsening, improvement, or no change in EDSS score during a 1-year period (52 weeks).

A decline in average daily step count occurred during the study period even when the EDSS score remained stable. Clinic-based measures did not change significantly from baseline to 1 year for many participants in this cohort of patients whose MS was well managed (Table). However, participants who experienced a clinically meaningful increase in disability during the study showed a decreased average daily step count (n = 21; β = −22.35; *P* < .001). Individuals with a decrease in EDSS score (corresponding to an improvement in function) did not have a statistically significant increase in average daily step count (n = 15; β = 5.74; *P* = .14). Individuals with no change in EDSS score during the year (stable disability) demonstrated a stable average daily step count over the year (n = 43; β = −1.15; *P* = .65) (Figure 2A). For participants with stable EDSS scores at study follow-up and who had a higher baseline average daily step count than participants in need of an assistive device to walk (EDSS score >5.5), there was a reduction in average daily step count (EDSS score, 0.0-1.5 [n = 7]; β = −20.75; *P* = .02; EDSS score, 2.0-3.5 [n = 12], β = −4.47; *P* = .41) and a small increase in average daily step count in 1 subgroup (EDSS score, 4.0-5.5 [n = 10]; β = 13.12; *P* = .004). For participants with EDSS scores of 6.0 and 6.5, there was a significant worsening in average daily step count during the study (EDSS score, 6.0 [n = 7]; β = −13.62; *P* < .001; EDSS score, 6.5 [n = 7]; β = −10.83; *P* < .001) (Figure 2B). Results were unchanged when considering individual Functional System Scores. A total of 51 of 90 patients (57%) demonstrated a difference in average daily step count of 800 steps from baseline (first 4 weeks) to 1 year (last 4 weeks): 33 of these 51 patients (65%) demonstrated a difference in average daily step count of more than 800 steps from baseline, and 18 of 51 patients (35%) demonstrated a difference in average daily step count of less than 800 steps from baseline.

### Worsening of Average Daily Step Count and Change in Outcomes

Patients with progressive MS had significantly fewer average daily steps during the year than did patients with relapsing MS (weighted mean [SD] for progressive MS, 4012 [3211] steps; for relapsing MS, 6388 [4373] steps; *P* < .001; unweighted mean [SD] for progressive MS, 3877 [2486] steps; for relapsing MS, 6066 [3319] steps; *P* = .001). There was no significant difference in the rate of change in average daily step count between the patients with relapsing MS and the patients with progressive MS (β = −4.09; *P* = .64, adjusting for sex, age, and disease duration).

**Figure 3.**
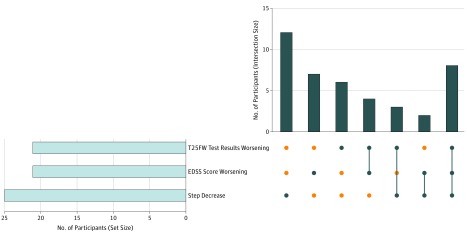
Number of Participants With Worsening in Expanded Disability Status Scale (EDSS) Score, Timed 25-Foot Walk (T25FW), or Average Daily Step Count During the 1-Year Study The horizontal bar graph to the left shows the number of participants who worsened in each of the 3 outcomes. The vertical bar graph to the right shows the shared number of participants (intersection size) who worsened during the year for 1 or more of the 3 outcomes, depicting each combination separately. A blue circle indicates whether that group of participants exhibited 1 or more of the 3 listed outcomes in the corresponding matrix cell. An orange circle indicates that that group of participants did not exhibit that outcome. A vertical blue line illustrates the column-based associations by indicating overlap (eg, a group with 2 blue circles connected by a vertical blue line exhibited both of those listed outcomes).

The EDSS score worsened for 21 of the 79 remaining participants (27%) at 1-year follow-up, 21 of 70 participants (30%) showed slower (worsening) T25FW times, and 25 of 79 participants (32%) showed a significant decrease in average daily step count during the year. These rates of disease progression during the study are most likely owing to the blocked recruitment study design and enrollment of participants across a range of ambulatory disability levels. Of note, 12 of 25 participants (48%) who showed a significant reduction in average daily step count did not experience either a decrease in T25FW or worsening of EDSS score (Figure 3), and similar trends were found when selecting only participants with progressive MS (19 of 49 [39%]) or relapsing MS (17 of 29 [59%]). A total of 7 of 21 participants (33%) with worsening EDSS scores did not experience worsening in either T25FW or average daily step count, and 6 of 21 participants (29%) with T25FW worsening did not experience either worsening of EDSS score or average daily step count. A total of 7 of 18 participants (39%) with a baseline EDSS score of 6.0 (using unilateral walking support) showed a significantly decreased average daily step count during the study but their clinic-based measures of disability (as measured by EDSS score) and walking speed (T25FW) did not reflect objective worsening.

**Figure 4.**
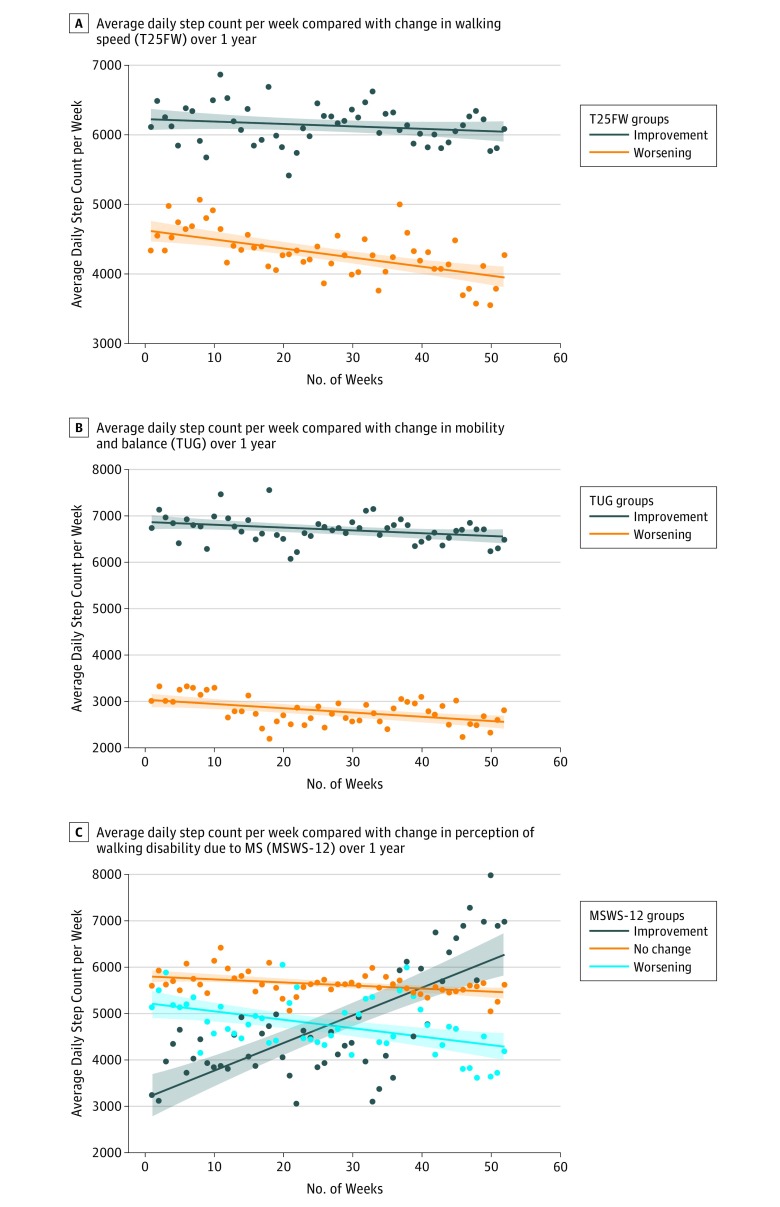
Average Daily Step Count per Week Compared With the Change in Clinic-Based and Patient-Reported Outcomes Each point reflects the average daily step count averaged per week (using weighted means) of individuals with changes in clinic-based and patient-reported outcomes from baseline to 1-year follow-up. The shaded areas represent the 95% CIs for the regression line. MSWS-12 indicates 12-item Multiple Sclerosis Walking Scale; TUG, Timed-Up-and-Go; and T25FW, Timed 25-Foot Walk.

Participants with worse T25FW at 1 year demonstrated a significant decrease in average daily step count (n = 21; β = −13.09; *P* < .001). Those with improved or stable walking speeds had no change in average daily step count (n = 44; β = −3.42; *P* = .19) (Figure 4A). Timed-Up-and-Go times worsened for participants who took fewer average daily steps during the study (β = −9.25; *P* < .001) (Figure 4B). For participants with stable TUG times, there was a significant decrease in average daily step count during the study (n = 42; β = −5.81; *P* = .03). Individuals who had a lower average daily step count during the study recorded longer TUG times at the 1-year follow-up (indicating decreased mobility and balance; n = 19; β = −9.25; *P* < .001).

The median daily step count for the entire cohort at baseline was 4766 (interquartile range, 2987-7448). No significant change in the average daily step count during the study was observed (mean [SD] of 315 [1632] fewer steps per day; *P* = .12), nor was there a significant difference in the rate of change in the average daily step count per week during the study period (β = −2.87; *P* = .14). However, the average daily step count significantly decreased during the study for participants with baseline EDSS scores of 0 to 1.5, 4.5 to 5.5, and 6.0 (EDSS score, 0.0-1.5 [n = 14], β = −33.94; *P* = .001 [corrected *P* = .01]; EDSS score, 4.5-5.5 [n = 12], β = −18.63; *P* = .001 [corrected *P* = .01]; EDSS score, 6.0 [n = 18], β = −7.89; *P* = .004 [corrected *P* = .01]). No difference in average daily step count during the year was determined in groups with an EDSS score of 2.0-3.5 (n = 23; β = 6.87; *P* = .14), EDSS score of 4.0 (n = 14; β = 4.03; *P* = .41), or EDSS score of 6.5 (n = 14; β = 0.68; *P* = .76) (Figure 2C). Results were unchanged after Bonferroni corrections.

A decrease in the average daily step count during the study did not appear to be associated with sex (β = −11.4; *P* = .19), mental health (Mental Health Inventory, β = −0.07; *P* = .33), or progressive MS (β = –11.3; *P* = .11) but was associated with relapsing MS (β = −15.4; *P* < .001). Only during month 12 was change in fatigue (Modified Fatigue Index) significantly associated with a decrease in the average daily step count during the study (OR, 0.84; 95% CI, 0.72-0.96; *P* = .02). No other time point or outcome (1.5, 3, 6, and 9 months) revealed significant associations. There was a significant association between average daily step count and individuals’ perception regarding the association of their MS on their walking. Individuals who improved (lower MSWS-12 scores) during the year demonstrated a significant association with increased average daily step count (n = 5; β = 59.93; *P* < .001). There was an association between decreased average daily step count and no meaningful change in MSWS-12 scores (n = 55; β = −6.97; *P* < .001). Subjective reports of greater association of MS affecting their walking (higher MSWS-12 score) were correlated with significantly fewer average daily steps taken during the year (n = 11; β = −17.96; *P* < .001) (Figure 4C). More important, there was a significant difference in slopes between shorter measurement epochs and the full 1-year data, specifically among people with ambulatory disability (EDSS score, >4), indicating that there is additional information to be gained from longer-term continuous monitoring (eTable 3 in the Supplement).

### Low Baseline Average Daily Step Count and Risk for Clinically Meaningful Worsening of MS

For participants whose baseline average daily step count was below the cohort median (4766), the odds of worsening disability (as measured by EDSS score) during the study was 4 times higher than for participants whose baseline average daily step count was above the cohort median (OR, 4.01; 95% CI, 1.17-13.78; *P* = .03), adjusting for age, sex, and disease duration (eTable 4 in the Supplement). This association between lower baseline average daily step count and increased odds of clinically meaningful worsening was also seen for other measures of ambulation, including the T25FW (OR, 3.50; 95% CI, 1.02-12.03; *P* = .05, adjusted for age, sex, and disease duration at baseline) and TUG (OR, 22.15; 95% CI, 5.40-152.62; *P* < .001).

## Discussion

### Continuous Activity Monitoring as an Ecologically Valid Assessment of Ambulatory Function in MS

We remotely monitored daily ambulatory activity during this 1-year study of individuals with MS with a wide range of ambulatory disability. One potential advantage of the average daily step count compared with clinic-based assessments is apparent in its face validity: the average daily step count measures actual daily ambulation, whereas metrics such as the EDSS, T25FW, and TUG are clinic-based proxies, and the MSWS-12 must rely on a patient’s memory and perception at the moment of completion. The average daily step count correlated well with these established, validated rater-based and patient-reported disability outcome measures, supporting its consideration as an ecologically valid outcome of daily physical function.

That retention was more than 80% at 1 year in our prospective cohort study is notable considering there was no additional incentive (other than perhaps novelty and utility) to wear the accelerometer. Previous efforts at characterizing physical activity were limited to approximately 7 to 10 consecutive days of monitoring (in some studies, this was repeated every 6 months, for up to 2 years).^25,26^ Moreover, most studies used research-grade accelerometers that are impractical for daily use over the longer term. The advantage of wrist-worn accelerometers is the unobtrusive acquisition of information about physical activity, and many patients already elect to use wrist-worn accelerometers for fitness tracking. This study demonstrates that data acquired from such devices can be clinically useful.

Although there was a modest reduction in the number of people providing valid accelerometer data (≥1 week per month) during the study, as depicted in Figure 1A, adherence to and use of the accelerometer was higher than might be expected from surveying the literature in the general population^27,28^ or among individuals with MS.^29^ Proactive study management strategies might account for the relatively favorable retention rate in our study. It will be important for future studies to optimize monitoring strategies and retention for this critical subset of patients with MS. The identification of strategies that may help ensure continued use of the tracking device is another strength of our study.

Optimal monitoring paradigms for the average daily step count need to be developed to minimize patient burden and maximize clinically meaningful information. Ideal models are likely to vary depending on the scientific question, patient demographics, and clinical context. Our data show that shorter monitoring epochs of the average daily step count are volatile and do not provide the same data as continuous year-long monitoring of patients with MS with ambulatory disability and that these additional data may be clinically meaningful.

### Average Daily Step Count as a Sensitive Measure of Ambulatory Function

Although a reduction in the average daily step count was associated with worsening on standard clinic-based and patient-reported metrics, the average daily step count may also capture aspects of function separate from and complementary to these measures. Imperfect associations and worsening of the average daily step count even when these standard metrics remained stable warrant further investigation to confirm the hypothesized clinical utility of the average daily step count (and exclude the potential that what the average daily step count is measuring in these cases may be clinically insignificant or erroneous). This finding is highlighted by the relatively high percentage of participants (7 of 18 [39%]) with a baseline EDSS score of 6.0 (using unilateral walking support), who showed a significantly decreased average daily step count during the study but whose clinic-based measures of disability (as measured by EDSS score) and walking speed (T25FW) did not reflect objective worsening. That the average daily step count appears likely to be more sensitive than either the EDSS or the T25FW at detecting clinically meaningful changes in ambulatory function in such patients could be leveraged to advance studies in progressive forms of MS and for potential neuroprotective and reparative therapies, in which early changes may be modest and may not be captured by current disability metrics alone. Although the average daily step count decreased in most subgroups, the lack of change in the average daily step count in the group with EDSS scores of 2.0 to 4.0 likely reflects the relative clinical stability of our clinic-based cohort receiving standard-of-care treatment with few relapses and potentially less risk of silent or overt clinical progression compared with those with higher baseline disability from MS.

### Average Daily Step Count as a Clinical Red Flag to Enrich Clinical Trials

A low-baseline average daily step count (defined for this analysis relative to our cohort median) was associated with a measurable increase in the risk of disability worsening at 1 year—a metric that, with further validation, could be clinically actionable and inform controlled trial designs. Specifically, screening study participants based on a low average daily step count has the potential to enrich trials by identifying individuals at highest risk of worsening disability during the near term, thereby reducing sample size, cost, and study time. A low average daily step count could also help inform clinicians when their patients may be at higher risk of near-term disability worsening and prompt interventions to prevent further progression.

### MS-Related Symptoms, Exercise, and Average Daily Step Count

Exercise has many well-established benefits for the health of people with MS.^30,31,32^ The average daily step count observed in this study was well below targets commonly cited as benchmarks for the general population.^32^ Prior studies report that people with MS have lower physical activity levels compared with sex- and age-matched controls.^33^ The data from our study could help inform efforts to promote increased physical activity among people with MS, such as individualized behavioral interventions by remote monitoring with a closed feedback loop regarding activity levels to optimize motivation and adjust goals.

The literature on longitudinal physical activity changes among patients with MS is limited,^25,26^ and variable results could lead to conflicting interpretations.^33,34,35,36^ The field has also tended to provide incomplete phenotypes and use subjective rather than objective measures for validation.^24^ Our study provides a framework for studying continuous activity monitoring using a widely accessible, commercial device during a full year in a cohort of patients with MS with disability assessments performed by interprofessional health care professionals (MS neurologist and a physical therapist). Continuous physical activity monitoring could be useful for monitoring patients with MS in both research and clinical settings. Moreover, individuals with other neurologic disorders in which ambulatory function is compromised, such as stroke, amyotrophic lateral sclerosis, movement disorders, and dementia, might also benefit from using the average daily step count as an outcome.

### Limitations

This study has some limitations. First, as is common in this emerging field, we calculated the average daily step count. There may be additional information encoded in smaller epoch (hour by hour or minute by minute) data or through defining bouts of activity that could benefit from future study as well as through machine learning approaches.^37,38^ Second, there is always potential for bias with missing data, especially with patients who are sick. There were times when participants did not wear their accelerometer for the entire day or for many days consecutively, although by collecting data continuously during the year, we were able to analyze the overall trajectory of physical activity. In addition, by defining a minimum threshold of the average daily step count for valid data, we may have removed extreme data points that were in fact informative about severe disability. Third, larger cohorts, and particularly study of the average daily step count in clinical trials, will be needed to determine the minimal epoch required to detect a change in ambulatory activity among people with MS undergoing a therapeutic intervention. Fourth, this study used a device that is already being supplanted by newer devices. Technology is changing at a fast pace. Studies exploring remote activity monitoring will need to develop strategies for comparing data across generations of devices. Fifth, there may be additional value in determining the association of the average daily step count with quantitative magnetic resonance imaging findings (particularly spinal cord metrics) currently used as surrogates for disability progression in individuals with MS, as well as other metrics of motor function and MS-related disability (including Functional System Scores associations); further studies are ongoing to measure and assess such associations.

## Conclusions

Continuous remote activity monitoring of patients with MS using a wrist-worn accelerometer during a 1-year period is feasible and reveals clinically relevant ambulatory disability not captured by standard metrics. These results appear to support the average daily step count as a sensitive and ecologically valid, longitudinal outcome measure in MS. Moreover, the average daily step count could be relevant for targeted intervention in controlled trials as well as in clinical practice in MS and other neurologic disorders.

## Notes

Supplement. **eTable 1.** Demographic Information for All Participants **eTable 2.** Demographic Table of Withdrawn or Lost to Follow-up Compared With the Completer Cohort **eTable 3**. Comparison of Shorter Epochs of Remote Average Daily Step Count to Continuous 1-Year Monitoring in People Without Ambulatory Disability (EDSS < 4.0) and People With Ambulatory Disability (EDSS ≥ 4.0) **eTable 4.** Odds of Disability Worsening at 1 Year in a Multivariable Logistic Regression Model
