# Supplementary material for: Association of Continuous Assessment of Step Count by Remote Monitoring With Disability Progression Among Adults With Multiple Sclerosis
Source: JAMA Netw Open. 2019 Mar 15;2(3):e190570. doi: 10.1001/jamanetworkopen.2019.0570 (PMC6484622; doi:10.1001/jamanetworkopen.2019.0570)
Supplement: Supplement. — eTable 1. Demographic Information for All Participants eTable 2. Demographic Table of Withdrawn or Lost to Follow-up Compared With the Completer Cohort eTable 3. Comparison of Shorter Epochs of Remote Average Daily Step Count to Continuous 1-Year Monitoring in People Without Ambulatory Disability (EDSS < 4.0) and People With Ambulatory Disability (EDSS ≥ 4.0) eTable 4. Odds of Disability Worsening at 1 Year in a Multivariable Logistic Regression Model [file jamanetwopen-2-e190570-s001.pdf]

Supplementary Online Content

Block VJ, Bove R, Zhao C, et al. Association of continuous assessment of step count by remote monitoring with disability progression among adults with multiple sclerosis. *JAMA Netw Open.* 2019;2(3):e190570. doi:10.1001/jamanetworkopen.2019.0570

eTable 1. Demographic Information for All Participants

eTable 2. Demographic Table of Withdrawn or Lost to Follow-up Compared With the Completer Cohort

eTable 3. Comparison of Shorter Epochs of Remote Average Daily Step Count to Continuous 1-Year Monitoring in People Without Ambulatory Disability (EDSS < 4.0) and People With Ambulatory Disability (EDSS ≥ 4.0)

eTable 4. Odds of Disability Worsening at 1 Year in a Multivariable Logistic Regression Model

This supplementary material has been provided by the authors to give readers additional information about their work.

© 2019 Block VJ et al. *JAMA Network Open.*

eTable 1. Demographic Information for All Participants

Demographic	Total	EDSS 0-1.5	EDSS 2-3.5	EDSS 4.0	EDSS 4.5-5.5	EDSS 6.0	EDSS 6.5
**Study Sample**	95	14	23	14	12	18	14
**Sex,** Female. N (%)	59 (62.1)	9 (15.3)*	17 (28.8)*	5 (8.5)*	7 (11.9)*	14 (23.7)*	7 (11.9)*
**Age (years),** mean (SD)	49.6 (13.6)	38.7 (13.2)	43.1 (11.5)	57.3 (11.7)	53.7 (9.6)	53.3 (11.1)	58.5 (13.2)
**Disease Duration (years)**, median [IQR]	13 [5.0- 20.5]	4.6 [3.0-8.0]	7.4 [2.9-14.5]	20.5 [9.9-30.9]	12.7 [8.7-15.3]	14.8 [5.6-22.9]	20.5 [14.6-22]
**Relapsing MS** (N)	60	14	20	7	6	9	7
**Progressive MS** (N)	35	0	3	7	6	9	7

Legend: MS = multiple sclerosis, EDSS = expanded disability status scale, N = number, SD = standard deviation, IQR = inter quartile range

* = percentages calculated using total Female as denominator (N=59)

© 2019 Block VJ et al. *JAMA Network Open.*

eTable 2. Demographic Table of Withdrawn or Lost to Follow-up Compared With the Completer Cohort

**Demographics and Outcomes**	**Completer Cohort (n=79)**	**Withdrawn or lost to follow-up (n=16)**	**Total (n=95)**	***P* Value (chi^2)**
**Female.** N (%)<br>**Male.** N (%)	30 (38.0)<br>49 (62.0)	6 (37.5)<br>10 (62.5)	36 (37.9)<br>59 (62.1)	.97<br>(.001)
**EDSS.** Median [IQR]	4.0 [2.5, 6.0]	3.5 [2.4, 4.9]	4.0 [2.5, 6.0]	.275
**Age.** Mean (SD)	50.3 (13.7)	45.9 (12.6)	49.6 (13.6)	.237
**Disease Duration (years)** Median [IQR]	13.0 [5.5, 20.5]	8.0 [3.0, 16.8]	13.0 [5.0, 20.5]	.620
**MS subtype.** RR: N (%), Prog: N (%)	49 (62),<br>30 (38)	11 (69),<br>5 (31)	60 (63),<br>35 (37)	.82<br>(.05)
**T25FW.** Mean (SD)	7.3 (5.8)	7.4 (5.0)	7.3 (5.6)	.922
**TUG.** Mean (SD)	12.2 (12.0)	10.8 (6.0)	11.9 (11.2)	.650
**MSWS-12.** Mean (SD)	36.2 (15.5)	36.1 (15.7)	36.2 (15.4)	.983
**MFIS-5.** Mean (SD)	9.9 (4.9)	9.8 (5.8)	9.9 (5.0)	.958
**MHI-5.** Mean (SD)	20.4 (2.1)	19.6 (3.1)	20.2 (2.3)	.249
**BLCS.** Mean (SD)	6.0 (5.9)	5.2 (6.7)	5.9 (6.0)	.660
**PES.** Mean (SD)	13.1 (5.6)	16.1 (6.2)	13.6 (5.8)	.057
**WHODAS.** Mean (SD)	10.4 (7.5)	12.4 (9.3)	10.7 (7.8)	.357

Legend: EDSS = Expanded disability status scale, IQR = interquartile range, SD = standard deviation, N/A = not applicable.

T25FW = Timed-25-Foot walk, TUG = Timed-up and Go, MSWS-12 = 12-item MS walking scale, MFIS-5 = 5 item Modified Fatigue Impact Scale, MHI-5 = 5-item Mental health Inventory, BLCS = Bladder control scale, PES = pain effects scales, WHODAS = World Health Organization Disability Assessment Schedule.

© 2019 Block VJ et al. *JAMA Network Open.*

eTable 3. Comparison of Shorter Epochs of Remote Average Daily Step Count to Continuous 1-Year Monitoring in People Without Ambulatory Disability (EDSS < 4.0) and People With Ambulatory Disability (EDSS ≥ 4.0)

**Slope of continuous average daily step count monitoring for 1 year compared to the slope derived from:**	**EDSS < 4.0 (slope of 1 year = - 9.34)**		**EDSS ≥ 4.0 (slope of 1 year = - 3.31)**	
	**Slope of shorter monitoring time**	**[*P* Value]**	**Slope of shorter monitoring time**	**[*P* Value]**
**Weeks 1 to 3**	330.21	.005*	125.33	.000**
**Weeks 1 to 4**	186.32	.048*	96.65	.000 **
**Weeks 1 to 5**	-96.42	.615	117.47	.000 **
**…**				
**Weeks 1 to 31**	-4.11	.594	-11.64	.021*
**Weeks 1 to 32**	-3.71	.545	-10.27	.048*
**Weeks 1 to 33**	-3.66	.520	-8.09	.184
**…**				
**First 2 weeks (1-2), and last 2 weeks (51-52)**	-13.13	.616	2.31	.056
**Baseline (weeks 1-4), month 6 and month 12.**	-13.21	.615	2.35	.096

Legend: EDSS = Expanded Disability Status Scale.

Significance: * p <.05, ** p <.001

To determine how shorter epochs of remote average daily step count compare to continuous monitoring for 1-year, we analyzed the difference in slopes for shorter durations, systematically eliminating one month at a time, compared to 1-year data. We dichotomized by EDSS to account for walking disability: participants with some walking disability (EDSS ≥ 4.0) and participants with minimal-to-no walking disability (EDSS<4.0). Shown in the table are the weeks where there was a transition to or from significance between the slopes.

© 2019 Block VJ et al. *JAMA Network Open.*

eTable 4. Odds of Disability Worsening at 1 Year in a Multivariable Logistic Regression Model

Outcome or Demographic	Odds Ratio	Confidence Interval	*P* Value
**EDSS Clinically Meaningful Worsening†**			
Median Average Daily Step Count at Baseline < 4766	4.01	1.17 – 13.78	.03
Age at Baseline (years)	2.45	0.81 – 7.43	.11
Sex (Male)	0.97	0.93 – 1.02	.21
Disease Duration	0.98	0.92 – 1.04	.47
**Timed-25 Foot Walk Worsening†**			
Median Average Daily Step Count at Baseline < 4766	3.50	1.02 – 12.03	.04
Age at Baseline (years)	3.02	0.94 – 9.69	.06
Sex (Male)	0.99	0.94 – 1.04	.64
Disease Duration (years)	1.01	0.95 – 1.07	.76

Legend: EDSS = Expanded Disability Status Scale.

† Clinically-meaningful disability (EDSS) change was defined as: a 1.5 point change when baseline EDSS score was 0.0 to 1.0; a 1.0 point change when baseline EDSS score was 1.5 to 5.0; and a 0.5 point change when baseline EDSS score was 5.5 to 6.5. A 20% change was considered clinically meaningful for T25FW.
Significance p values: P<.05. Adjusting for disease duration did not significantly alter the results.

© 2019 Block VJ et al. *JAMA Network Open.*
